# Influence of Trap Color, Type, and Placement on Capture Efficacy for *Protaetia brevitarsis* (Coleoptera: Scarabaeidae)

**DOI:** 10.1093/jee/toaa259

**Published:** 2020-12-09

**Authors:** Huanhuan Cai, Tao Zhang, Yinghua Su, Zhongyue Wang, Xiaofang Zhang, Shaoshan Wang, Yongqiang Liu

**Affiliations:** 1 College of Agriculture, Shihezi University, Shihezi, China; 2 Institute of Plant Protection, Hebei Academy of Agriculture and Forestry Sciences/Integrated Pest Management Center of Hebei Province/Key Laboratory of IPM on Crops in Northern Region of North China, Ministry of Agriculture, Baoding, China; 3 State Key Laboratory of Crop Biology, College of Life Sciences, Shandong Agricultural University, Tai’an, Shandong, China; 4 State Key Laboratory for Biology of Plant Diseases and Insect Pests, Institute of Plant Protection, Chinese Academy of Agricultural Sciences, Beijing, China

**Keywords:** *Protaetia brevitarsis*, trap characteristics, trapping location, trap color

## Abstract

In recent years, *Protaetia brevitarsis* Lewis has gradually become an important pest of several crops including grape (*Vitis vinifera* L.) and peach (*Amygdalus persica* L.) in Xinjiang, China. Toward improving trapping efficacy as part of a management program, various colors, types, and placement of traps and the use of an attractant were evaluated in field and laboratory studies. Laboratory color-choice tests and field tests indicated that *P. brevitarsis* adults preferred red. In trap placement tests, more adults were captured on traps placed 1 or 1.5 m above the ground and on top of the horizontal grape canopy. Before grape ripening, more adults were captured in traps placed in a 0.5-m border around the outside edge of the vineyard; during grape ripening, more were caught within the vineyard. Newly designed traps that were red, with a triangular baffle and a landing plate, were more efficient than traditional bucket-shaped traps. When *P. brevitarsis* adults were trapped and killed from June to July 2018, the population of *P. brevitarsis* adults in August to early September 2018 was significantly lower than in August to early September 2017, when adults had not been trapped and killed in the prior 2 mo.

*Protaetia brevitarsis* Lewis (Coleoptera: Scarabaeidae) is a serious pest of many fruit and vegetable crops, such as grape [*Vitis vinifera* L. (Vitaceae: Vitis)], peach [*Amygdalus persica* L. (Rosace: Pyrus)], and tomato [*Solanum lycopersicum* L. (Solanaceae: Lycopersicon)] in Mongolia, Japan, Russia, Korea, Northeast China and North China (Li et al. 2010, Suo et al. 2015). In Xinjiang, China, livestock manure in the unique mixed agriculture-pasture areas provide an ideal breeding ground for larvae of this pest, and fruit crops provide abundant food for adults, which have contributed to the emergence of *P. brevitarsis* adults as an important pest of fruit crops (Wang et al. 2011).

*Protaetia brevitarsis* adults feed on ripe fruits, and the peak in its adult population coincides with the ripening of their host plants, when chemical insecticides cannot be sprayed to ensure that no chemical residues are on fruits that will be consumed (Xu et al. 2009). In recent years, the most common controls have been catching and trapping of adults by bucket-shaped traps (75-mm diameter, 150- to 180-mm high) containing a mixture of sugar and acetic acid, but these techniques are not adequate, because the labor involved in such capture is expensive, and trapping with sugar–acetic acid does remove a sufficient proportion of the adult population of *P. brevitarsis* (Hao et al. 2005, Wang et al. 2011). Therefore, novel control strategies such as chemical communication-based trapping and killing have been developed against *P. brevitarsis* adults. Zhang et al. (2017) found that *P. brevitarsis* adults were significantly attracted to the compound X-ph-OR; however, the traps still need to be optimized, and factors that influence capture efficacy—trap color, type, and placement—need to be evaluated.

Insects not only use the odor of plant volatiles to identify hosts for feeding and laying eggs, but they also locate their hosts by visual signals, primarily color (Schoonhoven et al. 2005). For some insects, both olfaction and vision are involved in locating their host. For example, volatile odorants from plants are important cues for host selection by plant bugs (Hemiptera: Miridae) (Wheeler 2001) and in color-card trapping experiments with *Lygus lineolaris* (Palisot de Beauvois) (Hemiptera: Miridae), visual stimuli also play a role in host selection (Prokopy et al. 1979). Some insects such as *Empoasca fabae* (Harris) (Homoptera: Cicadellidae) rely on vision rather than olfaction in host searching (Bullas-Appleton et al. 2004), whereas others such as *Altica engstroemi* (Sahlberg) (Coleoptera: Chrysomelidae) locate their host plants mainly by vision.

The position of traps in an agroecosystem can also be important. More *Monochamus alternatus* (Hope) (Coleoptera: Cerambycidae) are captured on traps placed 1.5 m above the ground than at ground level or 3-m high (Ma et al. 2018). In a study of the efficacy of placement (exposed and shaded by plants) of sticky traps, more Cicindelidae were trapped in exposed traps, while shaded traps captured more Staphylinidae, Dolichopodidae, Cicadellidae, and Thripidae (Hoback et al. 1999).

To improve the efficacy of traps for capturing adult *P. brevitarsis* in integrated pest control programs, we thus tested trap color, type, and placement location to develop the most efficient trapping method for controlling the pest.

## Materials and Methods

### Experimental Field Sites

Experiments were carried out in a 2-ha vineyard in Yaer town, Gaochang district, Turpan city, Xinjiang Uygur Autonomous Region (42.95° N, 89.16° E). A 0.13-ha plot was used to assess the attractiveness of selected colors to *P. brevitarsis* adults, a 0.2-ha plot was used for the experiment on trap placement, a 0.13-ha plot was used for the experiment on trap type, and a 1.33-ha plot was used for the experiment on control efficacy. The grape variety Thompson Seedless (*Vitis vinifera* L.) matures in late July, and harvest continues through late August. The vines have been cultivated using a pergola system since 2003 with a row spacing of 1 × 5 m, and the horizontal canopy is 1.5 m above the ground. About 0.67 ha of peach, apricot [*Armeniaca vulgaris* L.(Rosace: apricot)) , and plum [*Prunus salicina* L. (Rosace: Prunus)] trees grow on the west side of the vineyard.Roads about 3-m wide are on the East, North, and South sides, and vineyards are across the road. No pesticides were applied during the experiment. At each trap location for all the experiments, a 2.2-m piece of a bamboo pole (1.9-cm diameter) was driven into the ground so that the top of the pole was ca. 2 m above ground level; traps were hung from the bamboo pole with wire at the height needed.

### Color Choice Assay

**Fig. 1.**
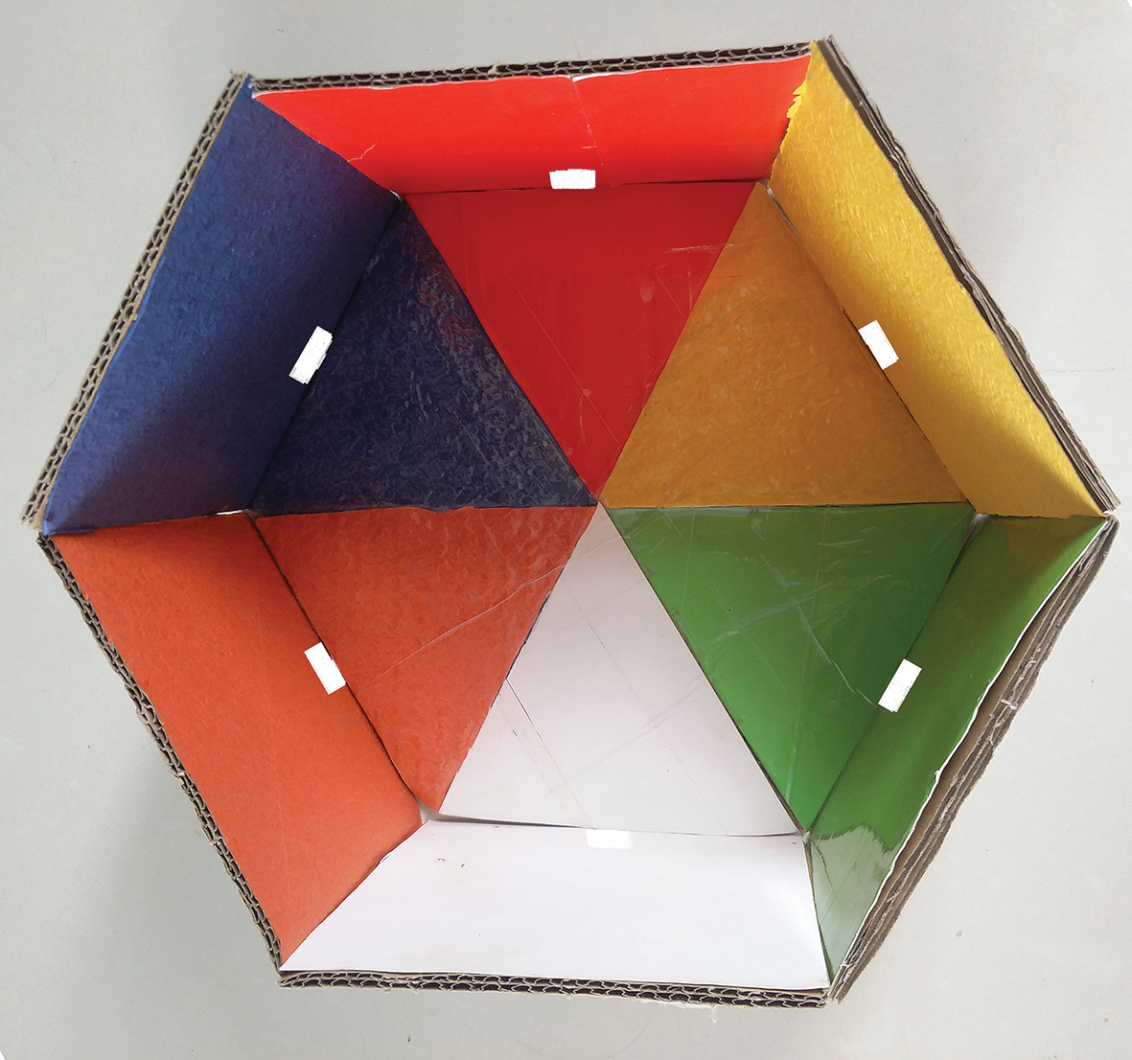
Hexagonal box for test colors for attracting adults of *Protaetia brevitarsis*.

A 15-cm high hexagonal box was constructed from cardboard (Fig. 1) with the bottom edge 15-cm long and an opening 3 × 2 cm in the middle of the lower part of each side. Paper in one of the test colors (red [RGB: 246, 64, 5], yellow [RGB: 229, 185, 78], blue [RGB: 66, 83, 129], green [RGB: 106, 128, 53], orange [RGB: 209, 85, 57], or white [RGB: 236, 235, 240]; the proportion of red, green, and blue [RGB] values were extracted by Photoshop software from a digital photo of the colored paper) was pasted on each interior side of the hexagon box and to the abutting bottom portion as shown in Fig. 1. The light source used in the experiment was natural light. An adult of *P. brevitarsis* was placed in the middle of the box and held in a transparent cup for 3 min to allow time for the adult to see all directions and colors. The color in the direction that *P. brevitarsis* adult crawled out was regarded as the chosen color. For each color, 120 adults (60 females, 60 males) were tested, and the experiment was repeated three times. The adults were captured in the vineyard in bucket-shaped traps with aclure (slow-release plastic vial with body and mouth diameters of 1 and 0.5 cm, respectively; Beijing Zhongjie Sifang Bio-tech Co., Ltd) in Yaer town, Gaochang district, Turpan city, Xinjiang Uygur Autonomous Region. For all experiments, the lures containing the attractant (*P*-methylphenol) in the traps were provided by the Institute of Plant Protection, Hebei Academy of Agriculture and Forestry Sciences and suspended with a wire from the top of the inside of traps. The bucket-shaped traps (top half yellow, bottom half white) for all experiments were purchased from Beijing Zhongjie Sifang Bio-tech Co., Ltd. Adults were immediately tested (about 2 h since them were captured) in the laboratory at 25 ± 2°C, 60 ± 5% relative humidity (RH).

### Field Verification of Red and Green Attractiveness to *P. brevitarsis* Adults

Because red was the color preferred by the adults in the laboratory test, we evaluated the effect of red on *P. brevitarsis* adults using the green background provided by plants as the control and pasted red (RGB: 246, 64, 51) or green (RGB: 106, 128, 53) papers on bucket-shaped traps. The trial was done in a completely randomized block design with nine replicate blocks, each block with one trap per placement ‘treatment’. Each block had a red trap and a green trap. Traps within blocks were placed 5 m apart in the vineyard and rotated weekly to randomize their position relative to the other trap, such that no trap of a particular color was placed in the same position twice during the experiment, and the closest trap to the vineyard border was 10 m from the border. Traps with the lure were placed 20 cm below the grape canopy on 20 July 2018, and trapped *P. brevitarsis* adults were counted on 3 August 2018, 14 d after the traps were set up.

### Trap Placement

The efficacy of trap placement for adult catches was tested in 2018 using the same bucket-shaped traps in three experiments with different trap placements: 1) 20 cm above and 20 cm below the grape canopy; 2) inside the vineyard and within 0.5 m of the edge of the vineyard; 3) 1.5, 1, or 0.5 m above the ground. Each experiment was done in a completely randomized block design with nine replicate blocks, each block with one trap per placement ‘treatment’. In experiment 1, each block had two traps, one above and one below the leaf curtain layer. There was 5 m between traps within and between blocks, and the closest trap to the vineyard border was 10 m from the border. Traps with lure were set out on 20 July, and trapped *P. brevitarsis* adults were counted 14 d later on 3 August. Experiment 2 was started before grape ripening and continued during ripening. Traps with lure were first set out on 1 June, and trapped *P. brevitarsis* adults were counted on 30 June. Traps with lure were set out again on 25 July, and trapped *P. brevitarsis* adults were counted on 25 August. The experiment had nine blocks, two placement treatments per block. Each block had two traps, one 0.5 m from the vineyard edge outside the vineyard perimeter, and the other 20 m from the vineyard edge inside the vineyard. Each block of traps was 5 m apart. Traps with the lure were placed 20 cm below the grape canopy. Experiment 3 also had nine blocks, with three placement treatments per block. In each block, a trap was placed 1.5, 1, and 0.5 m above the ground at the same location. Another block of traps was placed 5 m away, 0.5 m along the border of the vineyard. Traps with the lure were set out on 20 July, and trapped *P. brevitarsis* adults were counted 14 d later on 3 August.

### Trap Device

**Fig. 2.**
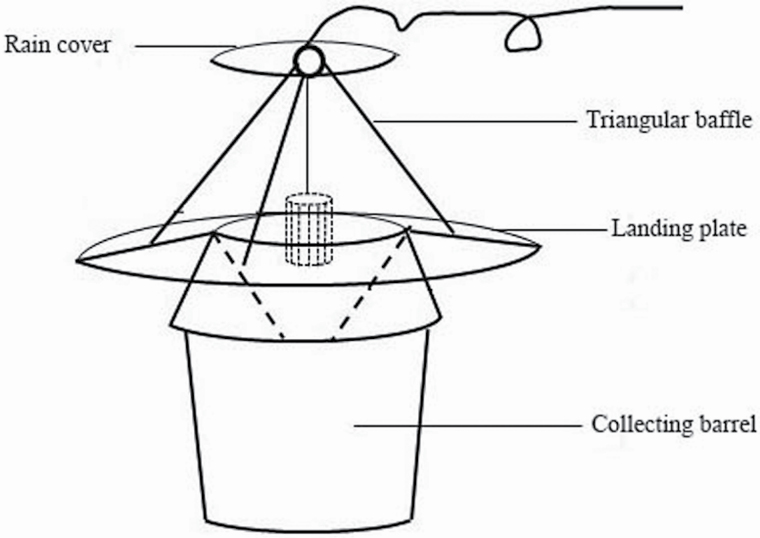
Newly designed red trap for adults of *Protaetia brevitarsis.*

A new trap was designed in red and consisted of four parts: a triangular baffle, a rain cover, a landing plate, and a collecting barrel (Fig. 2). The inclination angle of the landing plate was 5°, which can be used by adults for landing and to evacuate rainwater. The traditional traps were bucket-shaped traps. Each block a red trap and a green trap. The randomized complete block design consisted of nine blocks, each block with one trap per placement ‘treatment’. Two trap designs per block (the new trap and the traditional trap). Traps within each block were placed 5 m from each other and rotated weekly to randomize their position relative to other traps, such that no trap of a particular color was placed in the same position twice during the experiment, and the closest trap to the vineyard border was 10 m from the border. Traps with the lure were placed 20 cm below the grape canopy on 25 July 2018, and trapped *P. brevitarsis* adults were counted on 8 August 2018, 14 d after the traps were set out.

### Population Dynamics

The effect of trapping and killing *P. brevitarsis* adults in June and July 2018 on the population size in August and September 2018 was tested by comparing the number of adults trapped in August and September 2018 to the number trapped from 14 August to 2 October 2017, when adults were counted weekly in a survey in the vineyard in using the bucket-shaped traps with the lure. For both years, traps were set out 20 cm below the grape canopy, 20 m apart in a 1.33-ha vineyard, and 10 m from the vineyard edge; traps were set out a week before counting began. Lures were replaced monthly, and the trapped adults were removed weekly. The experiment consisted of three blocks with four traps per block. In 2018, *P. brevitarsis* adults were trapped and killed from 1 June to 31 July in 60 bucket-shaped traps with lure. From 14 August to 2 October 2018, adults were surveyed and counted as done in 2017.

### Data Analyses

All analyses were done using SPSS 13.0 (SPSS, Chicago, IL). Mean (±SE) proportion of *P. brevitarsis* adults attracted by different colors, mean (±SE) proportion of *P. brevitarsis* adults attracted by traps with different colors in field, at different locations (above and below the grape canopy and in a 0.5 m border around the outside edge of the vineyard and inside the vineyard), mean (±SE) proportion of *P. brevitarsis* adults attracted by different traps in the field, and mean (±SE) proportion of *P. brevitarsis* adults attracted by traps at three heights were analyzed separately using a one-way analysis of variance (ANOVA). If the ANOVA indicated a significant difference, Tukey’s honestly significant difference (HSD) test was then used to separate the means. Mean (±SE) proportion of *P. brevitarsis* attracted by different traps in the field and mean number of *P. brevitarsis* adults per trap in 2017–2018 were compared by Student *t* test. All proportion data were arcsine square-root-transformed before analysis (SPSS Incorporation 2006).

## Results

### Color Choice Assay

**Fig. 3.**
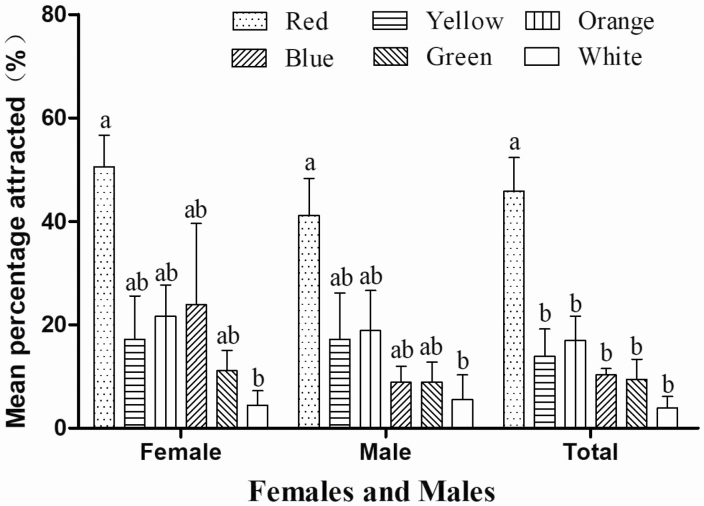
Mean (±SE) percentage of *Protaetia brevitarsis* attracted by different colors in the laboratory. Different letters above bars indicate a significant intermonth difference at the 5% level in Tukey’s HSD tests.

Color significantly affected attraction of *P. brevitarsis* adults. For females, red (mean ± SE: 50.56 ± 10.58%; *n* = 91) was the most attractive color and white (4.44 ± 4.81%; *n* = 4) the least attractive; attraction to blue (23.89 ± 27.15%; *n* = 21), orange (21.67 ± 10.41%; *n* = 27), yellow (17.22 ± 14.37%; *n* = 19), and green (11.11 ± 6.74%; *n* = 18) was intermediate (*F*_5, 12_ = 3.393, *P* = 0.038). For males, red (41.11 ± 12.51%; *n* = 74) was the most attractive color, white (5.55 ± 8.22%; *n* = 10) the least attractive; attraction to orange (18.89 ± 13.37%; *n* = 34), yellow (17.22 ± 15.49%; *n* = 31), blue (8.88 ± 5.36%; *n* = 16), and green (8.88 ± 6.74%; *n* = 16) was intermediate (*F*_5, 12_ = 3.589, *P* = 0.032). For both females and males, red (45.83 ± 11.21%; *n* = 165) was significantly more attractive than orange (16.94 ± 8.22%; *n* = 61), yellow (13.89 ± 9.22%; *n* = 50), blue (10.28 ± 2.10%; *n* = 37), green (9.44 ± 6.79%; *n* = 34), or white (3.89 ± 3.85%; *n* = 14; *F*_5, 12_ = 8.281, *P* = 0.001). Therefore, red was selected for the next field experiments (Fig. 3).

### Field Veriﬁcation of the Effectiveness of the Selected Color for Attraction of *P. brevitarsis* Adults

**Fig. 4.**
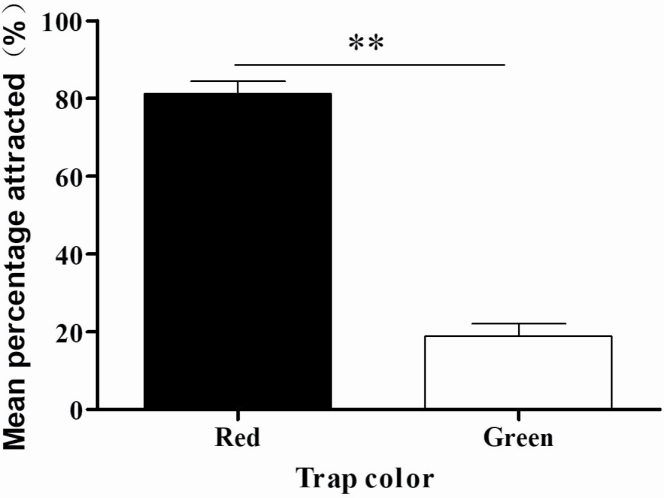
Mean (±SE) percentage of *Protaetia brevitarsis* attracted to red and green traps in the field. Double asterisks (**) indicate a significant difference at the 1% level (one-way ANOVA).

The color choice field experiment showed that significantly more adults were captured by traps in red (mean ± SE: 81.08 ± 8.55%; *n* = 209; *F*_1, 16_ = 174.596, *P* < 0.001; Fig. 4); therefore, red was chosen as the best trap color for *P. brevitarsis* adults.

### Trap Placement

**Fig. 5.**
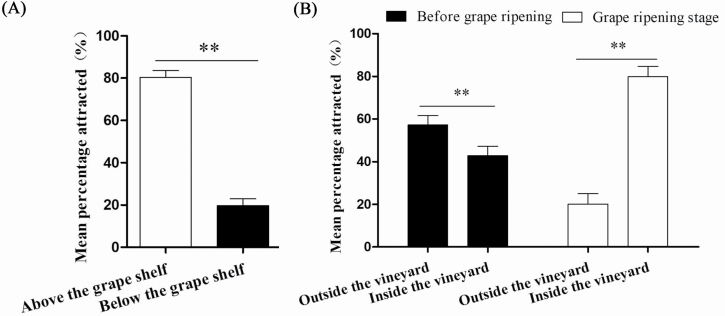
Mean (±SE) percentage of *Protaetia brevitarsis* attracted to traps above and below the grape horizontal canopy (A) or in 0.5 m border around the outside edge of the vineyard edge and inside the vineyard (B). Double asterisks (**) indicate a significant difference at the 1% level (one-way ANOVA).

Trap location had a significant effect on the number of *P. brevitarsis* adults captured. Significantly more *P. brevitarsis* adults were captured by traps above the grape canopy (mean ± SE: 80.36 ± 9.87%; *n* = 374) than below the grape canopy (19.64 ± 9.87%; *n* = 93; *F*_1, 16_ = 117.520, *P* < 0.001; Fig. 5A). Significantly more adults were captured by traps inside the vineyard (79.36 ± 10.91%; *n* = 293) than along the border of the vineyard (20.64 ± 10.91%; *n* = 75) from 25 July to 25 August during grape ripening (*F*_1, 16_ = 111.624, *P* < 0.001). Significantly more adults were captured by traps along the border of the vineyard (57.27 ± 9.32%; *n* = 453) than inside the vineyard (42.73 ± 9.32%; *n* = 292) from 1 to 30 June before grape ripening (*F*_1, 16_ = 6.147, *P* = 0.005; Fig. 5B).

**Fig. 6.**
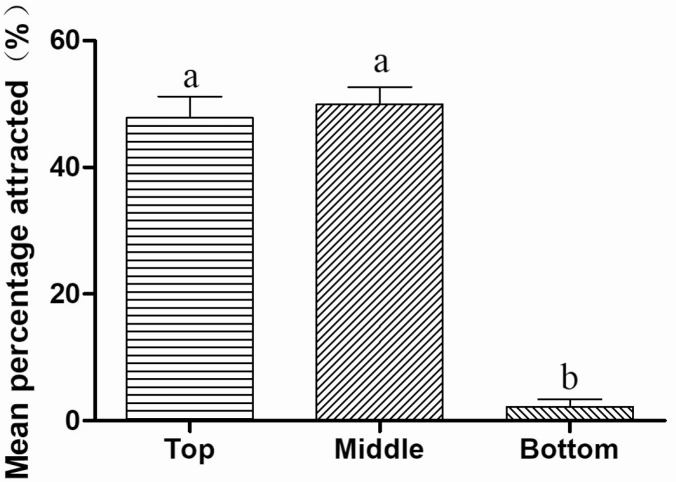
Mean (±SE) percentage of *Protaetia brevitarsis* attracted by traps placed 1.5 m above the ground, 1 m above the ground, or 0.5 m above the ground. Different letters above bars indicate a significant intermonth difference at the 5% level in Tukey’s HSD tests.

Trap height also had a significant effect on the number of *P. brevitarsis* adults captured. More adults were captured by traps at 1.5 and 1 m above the ground than at 0.5 m (1.5 m, 47.83 ± 10.01%, *n* = 150; 1 m, 49.93 ± 8.19%, *n* = 169; 0.5 m, 2.24 ± 3.48%, *n* = 11). The number caught at 1.5 and 1 m did not differ significantly (*F*_2, 24_ = 124.000, *P* < 0.001; Fig. 6).

### Trap Device

**Fig. 7.**
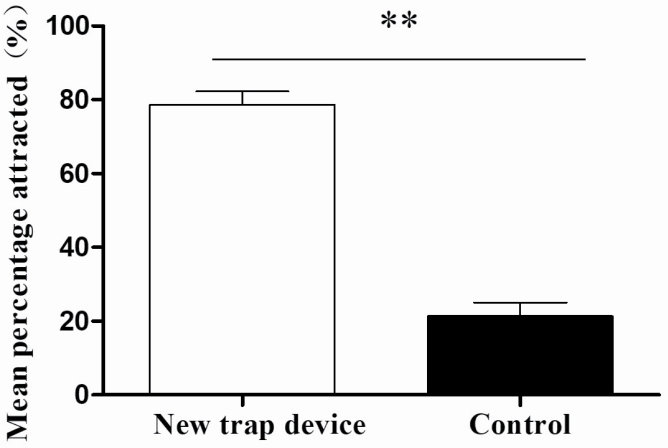
Mean (±SE) percentage of *Protaetia brevitarsis* attracted by the new trap (Fig. 2) and the traditional bucket trap in the field. Double asterisks (**) indicate a significant difference at the 1% level (one-way ANOVA).

Trap type had a significant effect on *P. brevitarsis* adult captures. The new traps (Fig. 2) caught more *P. brevitarsis* adults than control bucket-type traps (*F*_1, 16_ = 102.926, *P* < 0.001; Fig. 7). The mean percentage of total *P. brevitarsis* adults trapped by the new traps was 78.65 ± 11.05% (*n* = 1,430); 21.35 ± 11.05% (*n* = 351) were caught in the control bucket-type traps.

### Population Dynamics

**Fig. 8:**
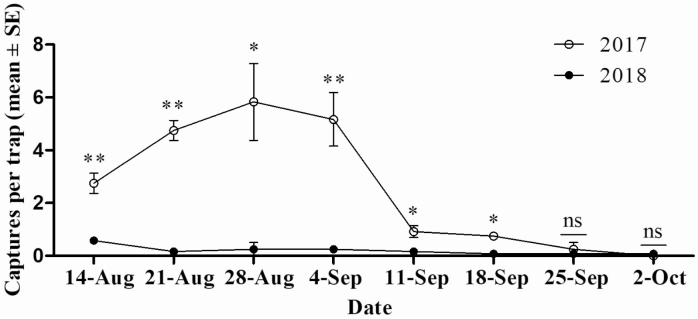
Mean number of adult *Protaetia brevitarsis* captured per trap in 2017–2018. Adults were trapped and killed in June to July 2018, but not in 2017. Single asterisk (*) or double asterisks (**) indicate a significant difference at the 5 or 1% level, respectively; ns indicates difference was not significant (one-way ANOVA).

The adult stage of *P. brevitarsis* in Xinjiang occurs from early June to early September (Xu et al. 2009). Our survey results also showed that the adult population began to decrease in early September and was very low in mid to late September, with an average of less than one specimen captured per trap. Figure 8 shows that significantly fewer *P. brevitarsis* adults were present in 2018 than in 2017 from mid-August to the end of August after adults were trapped and killed from June to July 2018 (14 August: *t* = 6.448, df = 4, *P* = 0.003; 21 August: *t* = 11.379, df = 4, *P* < 0.001; 28 August: *t* = 4.06, df = 4, *P* = 0.015; 4 September: *t* = 4.85, df = 4, *P* = 0.008; 11 September: *t* = 3.182, df = 4, *P* = 0.033; 18 September: *t* = 4.00, df = 4, *P* = 0.016; 25 September: *t* = 0.632, df = 4, *P* = 0.561; 2 October: *t* = 0.632, df = 4, *P* = 0.561).

## Discussion

Colors can be recognized by herbivores as visual signals for host location and ovipositioning (Mazzoni et al. 2009, Fred and Brommer 2010, Kühnle and Müller 2011). The color that is most attractive to an insect species may depend on its specific resources and environmental needs (Mizell and Tedders 1999). For example, the root weevil, *Sitona lepidus* (Gyllenhal) (Coleoptera: Curculionidae), is attracted to yellow (Cross et al. 1976, Riley and Schuster 1994). On the other hand, the root weevils *Hylobius pales* (Herbst) (Coleoptera: Curculionidae) and *Pachylobius picivorus* (Germar) (Coleoptera: Curculionidae) are more attracted to black and brown than to other colors (Mizell and Tedders 1999), and our laboratory and field experiments showed that *Pachylobius brevitarsis* adults preferred red. The root weevil prefers the color of the root environment such as yellow, black, and brown. *Pachylobius brevitarsis* adults prefer feeding on ripe fruits such as peach and grape, which are primarily red at maturity. Our results showed that *P. brevitarsis* adults preferred red, consistent with the fruit color of their favorite hosts.

Trapping location is another important factor that affects trap catches as demonstrated for *Cosmopolites sordidus* (Germar) (Coleoptera: Curculionidae) (Reddy et al. 2009), *Rhabdoscelus obscurus* (Boisduval) (Coleoptera: Curculionidae) (Reddy et al. 2011), *Limotettix vaccinia* (Van Duzee) (Hemiptera: Cicadellidae) and *Scaphytopius magdalensis* (Provancher) (Hemiptera: Cicadellidae) (Rodriguez-Saona et al. 2012). Various insects react differently to traps at various heights, perhaps reflecting differences in the resolution of their eyes or in their preferred flight height (Byers 2011). For *P. brevitarsis* adults, the most effective trap heights were 1 and 1.5 m above the ground. In addition, more adults were captured by traps above the grape canopy than below, suggesting that the *P. brevitarsis* adults like to fly above the grape canopy and that grape canopy might block their vision and flight.

We also found that more *P. brevitarsis* adults were captured by traps along the border of the vineyard than inside the vineyard before grape ripening, possibly because before grape ripening, food resources do not differ between the vineyard and the border. In addition, if there is no grape canopy blocking the vision and flight of *P. brevitarsis* adults along the border of the vineyard, the adults can more easily find the traps. But after ripening, more *P. brevitarsis* adults were captured by traps inside the vineyard than along the border of the vineyard; the ripening grapes likely provide abundant food for the adults, so more will be in the vineyard. Therefore, more adults are trapped along the border of the vineyard before grape ripening than after; after ripening, more adults are in the vineyard where traps will be most effective.

The design of traps will determine the aerodynamics and the efficacy of the trap. For example, window traps made of solid surfaces such as plastic or glass can cause insects in the air to be buffeted around the trap, whereas screen traps that allow air to pass through do not appear to have this problem (Byers 2011). Our results showed traps with a triangular baffle and landing plate are better at trapping of *P. brevitarsis* adults, perhaps the larger adults are not affected by the airflow, and the landing plate provides support for its landing. In the present study, *P. brevitarsis* adults were found to be most attracted to red and least attracted to white, so red and white used in the design of the trap. Red was used in the upper part of the trap with the entrance, where it is more convenient for entry of *P. brevitarsis* adults. White was used in the lower part of the trap (trap barrel) to reduce the attraction of *P. brevitarsis* adults by the lower part and increase the trapping effect of the upper part. Our results showed that newly designed traps were more efficient than traditional bucket-shaped traps. Therefore, the efficacy of the trap can be improved using the preferred color combined with an avoidance or nonresponsive color.

Although *P. brevitarsis* produces one generation a year, its adult stage in the area of Xinjiang lasts from early June to September, time enough to cause serious damage (Xu et al. 2009, Liu et al. 2012). Grape ripening is an important period for the damage of *P. brevitarsis* adults, and Thompson Seedless grapes, the main grape variety in Xinjiang, matures in late July. To evaluate the control efficacy of trapping and killing of *P. brevitarsis* adults before grape ripening on their population level during grape ripening, we trapped and killed the adults in June and July 2018 and compared the number of *P. brevitarsis* adults in August and September to the numbers in the same period the previous year when no early trapping and killing was done. The adult population in August–September 2018 was significantly lower than that in the same period in 2017. Thus, the evaluation of trap and kill in field trials should be studied for the establishment of *P. brevitarsis* adults control technology.

For controlling *P. brevitarsis* adults, control measures should be chosen according to adult habits and implemented during the key control period(s). Our results showed that *P. brevitarsis* adults preferred red, flying at an altitude over 1 m and above of the grape canopy and that the number of adults can be reduced in the tested vineyard by trapping and killing the *P. brevitarsis* adults in June and July. Therefore, using red as the main trap color and setting traps on the top of the shelf from June to September should greatly improve the control efficacy for adult of *P. brevitarsis* in vineyard in Xinjiang, China.
